# Activity-Aware Vital SignMonitoring Based on a Multi-Agent Architecture

**DOI:** 10.3390/s21124181

**Published:** 2021-06-18

**Authors:** Todor Ivașcu, Viorel Negru

**Affiliations:** Department of Computer Science, Faculty of Mathematics and Informatics, West University of Timisoara, Blvd. V. Pârvan nr. 4, 300223 Timișoara, Romania; viorel.negru@e-uvt.ro

**Keywords:** human activity recognition, vital signs, health status monitoring, wearable sensors, multi-agent architecture, knowledge-based system

## Abstract

Vital sign monitoring outside the clinical environment based on wearable sensors ensures better support in assessing a patient’s health condition, and in case of health deterioration, automatic alerts can be sent to the care providers. In everyday life, the users can perform different physical activities, and considering that vital sign measurements depend on the intensity of the activity, we proposed an architecture based on the multi-agent paradigm to handle this issue dynamically. Different types of agents were proposed that processed different sensor signals and recognized simple activities of daily living. The system was validated using a real-life dataset where subjects wore accelerometer sensors on the chest, wrist, and ankle. The system relied on ontology-based models to address the data heterogeneity and combined different wearable sensor sources in order to achieve better performance. The results showed an accuracy of 95.25% on intersubject activity classification. Moreover, the proposed method, which automatically extracted vital sign threshold ranges for each physical activity recognized by the system, showed promising results for remote health status evaluation.

## 1. Introduction

The world population is aging rapidly, and according to a World Health Organization (WHO) report [1], it is estimated that the number of elderly people over 60 years of age will double by 2050, reaching approximately 2.1 billion, with 80% of them living in developing countries. Chronic diseases are another issue that affects the quality of life of the population. Often, chronic disease patients require continuous monitoring, which increases the number of patient visits to the hospital. Another issue is that symptoms can be absent during hospital visits, and this makes it difficult for physicians to establish the correct diagnosis. To reduce the healthcare costs and to improve the quality of life, different remote, real-time, and continuous monitoring solutions have been proposed in recent years. With the recent development and advancement of wearable and portable sensing technologies, it is possible to continuously monitor patients’ health conditions and the effectiveness of exercise and treatments, outside the clinical environment, during daily life (at work, at home, during sports or fitness activities, etc.).

In the last few years, wearable sensors and wireless body area networks (WBANs) have been used in different medical and non-medical applications in order to monitor patients’ physiological measurements [2,3,4], physical activities [5,6], as well as for fall detection [7,8,9]. WBANs, which are a relatively new and emerging technology, first introduced by [10], consist of tiny body-attached sensors that can noninvasively monitor and measure physiological (vital) signs, such as the heart rate (HR), the respiratory/breathing rate (BR), blood pressure (BP), the blood oxygen level (SpO${}_{2}$), the electrocardiogram (ECG), etc., and are capable of sending data to other devices for further processing and analysis, where appropriate actions can be taken. The major role of such a system is to ensure better support in disease diagnosis or to alert medical services when a major deterioration has happened in the patients’ health status.

Changes in vital signs are an important indicator of health status deterioration, and generating alerts about the patients’ health condition to caretakers or medical personnel in case of critical situations is very useful in order to provide immediate help. In recent years, different remote monitoring systems were proposed that use a data fusion model for health status evaluation. These systems use the early warning score (EWS) [11] model, a simple physiological aggregate scoring system. Ranges for each vital sign are configured, and in most cases, based on fuzzy set theory and a decision matrix, alarms are generated when an emergency is detected.

Vital sign measurements are related to the intensity of the activity that the user performs; thus, physiological measurements increase in proportion to the increase in the intensity and metabolic needs of the activity [12,13,14,15,16,17,18,19,20,21,22]. Vital signs’ normal threshold ranges can be considered when a patient is sitting quietly. Inside and outside the clinical environment, where users can perform light-, moderate-, and vigorous-intensity activities, different threshold ranges must be considered as normal in order to not generate false alarms. To solve this issue, the monitoring system must recognize the patient’s physical activity. In this work, we took into account the metabolic equivalent of tasks (METs) [23], a measure that expresses the energy cost of each physical activity as a multiple of the resting metabolic rate. One MET, defined as 1 kcal/kg/hour, is considered when a person is resting, obtained while quietly sitting. A physical activity of three METs would require three-times the energy that an average person consumes while quietly sitting. Information about the physical activity is useful either for medical personnel when analyzing health data or for the system when generating alarms. On the other hand, in the majority of recent research work, in case of the deterioration of the user’s health status, the notifications or alarms are displayed to the user or sent to the caretakers/medical personnel based on the manual customization of the threshold ranges, either by the final user or by the medical personnel according to the user’s particularities, so an adaptability mechanism must be considered.

In recent years, human activity recognition (HAR) has been investigated using various types of sensors, smartphones [24,25], body-attached sensors [26,27], ambient sensors [28], video-based information [29], etc. Considering the privacy concerns of installing a camera in private spaces, sensor-based activity recognition has dominated the recent research. Furthermore, the advantage of using a smartphone or body-attached sensors for activity recognition is that the person can move freely, at home or at work, while being continuously monitored without dependence on the environment. In recent research work on human physical activities, different sensing modalities were considered, such as accelerometers, gyroscopes, and magnetometers; the optimal sensor position; the sampling rate; feature extraction and selection; and even using a single sensor or aggregating different sources [30,31,32,33,34]. Aggregating data from multiple sources has shown better classification accuracy. In order to recognize physical activities, different machine learning models were considered, such as the support vector machine (SVM), decision tree (DT), artificial neural network (ANN), random forest (RF), and deep learning (DL) approaches.

One of the challenges in WBAN technology, and in sensor networks in general, is the fusion of data from different heterogeneous sources, which would directly impact the performance of the application [35,36]. Different body-attached and wearable sensors come from different manufacturers; different types of physiological measurements can be recorded; different techniques of signal processing must be performed; recorded data can be collected at different time intervals; thus, considering the complexity of the system, the heterogeneity of hardware devices and data, and the necessity for flexibility, in this work, a multi-agent architecture was considered.

The key contributions of this research are the following:

- To build an activity recognition component that uses the accelerometer data obtained from different wearable sensors in order to discriminate between different activities of daily living;
- To automatically extract threshold ranges for different monitored vital signs, from the group of users when performing different physical activities, which are used for evaluating the health status of a target user;
- To implement a multi-agent architecture that is easily adaptable to the sensor network and hardware from different manufacturers, which can process signals from different body sensors, recognize the physical activity, evaluate the user’s health status based on the recognized activity, and alarm when necessary, using ontology-based models to address the data heterogeneity.

The rest of the paper is organized as follows. Section 2 summarizes the related studies in vital sign extraction, human activity recognition, multi-agent systems, and semantic modeling. In Section 3, we introduce the proposed multi-agent architecture and present the different system components and their behaviors. Results and a discussion regarding the activity recognition performance and the extracted knowledge about the vital sign ranges are presented in Section 4. Finally, the conclusions and future research directions are summarized in Section 5.

## 2. Background and Related Work

### 2.1. Vital Sign Monitoring

The electrocardiogram (ECG) is one of the most widely used biosignals to analyze cardiac rhythm. Different vital signs, such as the HR and BR, which are extremely valuable indicators of the user’s health condition, can be extracted from this signal. On the ECG waveform, a QRS complex is the most recognizable part. The most differentiated peak on the waveform is the R peak. The interval between two consecutive peaks (also known as the RR-interval) is used to determine the HR. For the ECG signal, a higher sampling rate is required, but it shows acceptable results using only 50 Hz [37,38].

In [39], the authors presented an algorithm for heart rate analysis from photoplethysmogram (PPG) and ECG signals collected in noisy settings. The algorithm was validated on two different datasets. On the ECG signal, the algorithm successfully detected 99.72% of the peaks present. In order to estimate the BR from the ECG signal, the RR-interval can be used. During inspiration, the heart rate increases, meaning a shorter RR-interval, and during expiration, this results in a longer RR-interval.

In [40], the authors discussed an algorithm for extracting ECG-derived respiration. Two methods were considered for extracting the BR, the heart rate variability (HRV) method and the peak amplitude variation (PAV) method, as well as interpolating the signal’s amplitude using cubic spline interpolation. The algorithm was analyzed on a database that included 30 subjects, and a mean absolute error (MAE) value of 0.57 and 0.70 for the HRV and PAV method, respectively, was obtained.

### 2.2. Physical Activity Recognition

Accelerometer-based activity recognition has been increasingly used in recent years because of the good classification results compared to other sensing modalities. In order to recognize physical activities from the accelerometer signal, several steps must be considered, which implies data sampling, data segmentation, feature extraction, the selection of the most discriminant features, and model training.

The literature review highlighted that the most common sampling rate used in human activity recognition is around 50Hz, which contains enough information for simple activities of daily living [41]. Segmentation techniques, which include different methods and window sizes, have shown an impact on the classification accuracy. The most used is the fixed-size nonoverlapping sliding window (FNSW) and the fixed-size overlapping sliding window (FOSW) [41,42]. In [43], the authors investigated the effectiveness of the window-based segmentation approach with different window sizes showing that the sliding window approach was generally effective at HAR. Usually, a window with a size that ranges between 1 s and 10 s is used [44]. Simple activities of daily living can be recognized with good accuracy with a window size as short as 2 s [45].

Feature extraction has an important role in activity classification. Features can be extracted from both the time domain and the frequency domain of the signal. In the literature, the most common metrics that were extracted from the signal data were: max, min, mean, median, variance, kurtosis, skewness, zero-crossing rate, root mean square, standard deviation, interquartile range, signal magnitude area, energy, and signal entropy [30,44]. Different positions of the sensors have been considered, usually placed on the subject’s chest, wrist, ankle, waist, hip, and thigh. The position of the sensors has an important role in the classifier’s accuracy. In [46], the authors investigated various methods for feature extraction from different accelerometer placements, achieving a 95% of classifier accuracy, applying the intersubject cross-validation method, using features from the time and frequency domains. By combining data from different sources, an improvement in the classification accuracy could be achieved. In [47], the authors reported an accuracy of 97.20% for eight daily living activities, by combining the data from seven wearable sensors applying the DL method. On the other hand, by combining the data only from two sensors, the best score obtained from the shin and forearm positions with 93% accuracy was reported.

The most used classifiers in the literature are artificial neural networks/multi-layer perceptron, random forest, decision trees (C4.5/J48), support vector machines, K-nearest neighbors, naive Bayes, and the hidden Markov model, which showed good classification of activities of daily living.

In [48], the authors presented a subject’s monitoring system based on IoT technology, which used a single wearable device, a Zephyr^TM^ Bioharness 3 Model K113045. The system implemented two modules: an activity recognition module, which could be implemented on any mobile device, and an e-health application, which could be implemented on the server. Two machine learning methods were implemented for the classifiers, a Bayesian algorithm (naive Bayes) and a decision tree (the C4.5 model), for activity recognition. Using a small dataset, an accuracy of 95.83% was achieved on four types of activities. An accuracy of 100% was reported in [49]. A model based on an RF classifier was used to recognize six activities of daily living, extracting the angle, maximum, minimum, and mean values from the accelerometer and gyroscope signals. These features were selected by their importance score. The subject-dependent hold-out method, by dividing the data randomly into 70% and 30% for training and testing, respectively, was applied.

### 2.3. Multi-Agent Systems

In recent years, due to their characteristics, such as autonomy, reactiveness, proactiveness, and social ability, agents have been widely used in different WBAN/IoT-based healthcare applications [50]. The scalability, heterogeneity, flexibility, and distributed nature are the major challenges that must be considered in these applications, and the multi-agent system (MAS) architecture has proven to be a very effective solution. The multi-agent distributed information platform (MADIP) system proposed in [51], composed of six types of intelligent agents, was one of the first e-health platforms based on multi-agent systems integrating remote and continuous vital sign monitoring, having a diagnosis component, automatically alerting care providers in case of physiological abnormalities, and also, offering remote medical advice.

In [52], the authors proposed a flexible MAS architecture with a clear separation of concerns. Agents’ activities were organized according to the different types of roles, represented as a group of related goals and tasks. Each agent adopted a role and pursued its goals, performed tasks, and adapted to the available resources and information. These activities were related to sensor management, signal processing, agents’ interaction, and adaptation to the changes in available sensor devices and their capabilities. Furthermore, agents can form teams, a hierarchical group of roles, performing collaborative activities in the platform. A MAS architecture, based on a virtual organization of agents, for remote patient monitoring, was presented in [53]. The system, which allowed the integration of different sensor devices, monitors the patient’s health condition by analyzing the ECG signal. A physical activity monitoring component was also implemented by using the accelerometer data. This component was used for detecting whether a patient was in motion or not. In this architecture, an agent with the ECG analysis role, in case of anomaly detection in the physiological signal, could send notifications or alerts automatically to the emergency services. The alert also included information about the user’s movements, which was very useful when medical personnel analyzed the data. In [54], the authors proposed an intelligent heart rate monitoring system based on agents. The system could collect vital signs, detect anomalies, and send alerts to relatives or medical personnel. The authors raised the self-adaptation and self-management issues, which were their motivations in using the agent-based approach by adding an agent to each element in the architecture.

In order to react adequately and to adapt to the monitored environment, the system must be aware of the context and its changes. In [55], the authors proposed solutions for the design, implementation, and management of home care applications for elderly people. The system analyzed different context information, patient health status, or environment conditions and adapted its behavior according to the relevant context changes, for example in which circumstances the monitored vital sign was out of range.

Context modeling allows the MAS to better understand the environment and the changes that were made to its environment. Knowledge modeling using ontologies has been regarded to be one of the best solutions for representing context information [56]. The MAS system must be aware of different concepts that characterize different entities, such as the user’s profile, the information in the databases, the sensor devices and their capabilities, the type of signal that is processed, or other situations. Therefore, using the specific domain ontologies and semantically describing these concepts allowed the system to perform better and to complete its tasks.

## 3. Proposed Architecture

**Figure 1:**
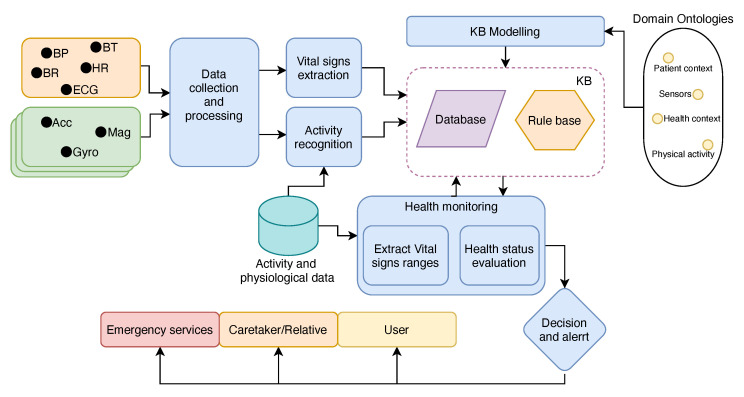
Proposed system’s workflow.

Before we detail the multi-agent architecture, we want to highlight the system’s main functionalities first. The system’s workflow can be seen in Figure 1. The proposed system was made of six main subsystems: the *data collection and processing subsystem*, which collects and processes the signals from various wearable/body-attached sensors; the *activity recognition subsystem*, which uses stored physical activity data to train different machine learning models in order to recognize the user’s physical activity and store that information in the knowledge base (KB); the *vital sign extraction subsystem*, which extracts physiological data from the given signal according to available algorithms/techniques and stores the measurements in the knowledge base; the *knowledge base modeling and management subsystem*, which uses and combines different domain-specific ontologies to model and manage the knowledge base; the *health monitoring subsystem*, which uses stored physiological data from different users to extract new knowledge from these data, by means of extracting monitored vital sign ranges, and evaluates user’s health status by monitoring vital sign measurements stored in the knowledge base; and the *decision and alert subsystem*, which takes the appropriate actions related to the user’s health condition and informing different external subsystems.

### 3.1. Knowledge Representation and Semantic Modeling

The knowledge of each agent, as well as about the monitored subjects, sensor devices, and health-related context is semantically represented using domain-specific ontologies. In this work, we used different existing and publicly available ontologies, as well as others defined/extended by us. The Vital Sign Ontology [57] was used to model the HR and BR data collected from the sensors. For the physical activity concepts, we did not find an ontology that was suited better to our requirements. The closest was the Physical Activity Concept Ontology (PACO) [58], but it did not cover all the concepts that we needed. Inspired by this ontology, we defined a simple ontology, using the open-source ontology editor framework Protege Version 5.1.0, which contains the activities of daily living concepts that were used in this work, the intensity of activities and the relation between activity, and their cost or metabolic equivalent score. For the sensor concepts, we integrated the Semantic Sensor Network Ontology [59], which was extended to represent the particularities of the sensors (IMUs) for activity recognition, their sensing modalities, the sampling rate, and the positions on the body. For this, we were inspired by the MIMU-Wear Ontology [60]. In addition to health sensors (the ECG sensor used in this work), the HealthIoT Ontology [61] was used as a model. We developed a patient context ontology that modeled the context about the patient by capturing different key information, such as the patient’s demographics, health status, relatives, or assigned physician, which were necessary for providing health care. An individual patient from this ontology was used to represent the monitored patient in the application. This individual was a central point in the knowledge base; all other concepts were related directly or indirectly to this one.

The developed/extended ontologies only strictly covered the most important concepts that were used in this work, and most of the inverse relations were omitted. The interontology concept mapping the relations among the user and sensors, the vital signs, and the activities, as well as the relation between the health sensor and the vital signs were realized manually. The previously mentioned ontologies were combined into a single one after which the knowledge base was modeled. Individuals from these ontologies were used to model the data and the relationships between the data in the knowledge base. The knowledge base acted as a blackboard where different system components could store their actual knowledge and be accessed/visualized by the interested parts.

### 3.2. MAS

**Figure 2:**
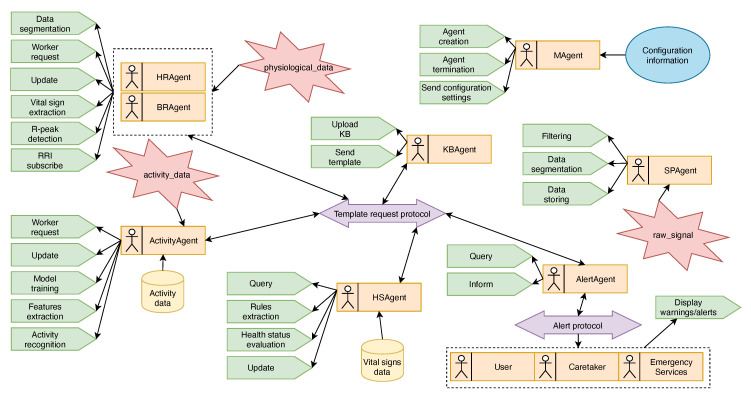
Proposed multi-agent architecture.

The proposed multi-agent architecture, which was an extension and completion of our previous work presented in [62], considered different types of specialized agents in the knowledge extraction, knowledge management, signal processing, and interaction with external systems. Some of these agents communicate directly with other agents, while some access shared information on the blackboard, which acted as a common shared service for all agents. The agents that were present in the proposed architecture are illustrated in Figure 2. Next, we describe the agent types and their roles and behaviors. Some basic agent interactions, such as initialization, registration to the directory facilitator (DF) services, etc., are also implied, but are not mentioned.

Manager agent (MAgent): A single instance of this type of agent exists in the monitoring platform. Its main task is to coordinate the MAS. It receives information or updates from the application about the monitored user, the sensors, and the types of sensors that are used for monitoring and initializes other agents that will take care of a specific task, or can form a team of agents, depending on the monitoring scenario.

Knowledge base agent (KBAgent): Just as in the case of the Manager agent, a single instance of this type of agent exists in the monitoring platform. Its main task is to manage the knowledge base. It selects and loads the predefined domain-specific ontologies in order to model the KB. Because the KB location, the name of the classes, or individuals, and properties can change over time, it holds the knowledge of the structure of the KB and the form of the SPARQL queries. It receives information from the Manager agent about the user and sensors and creates data individuals. Furthermore, this agent stores and labels the datasets that are used by other agents to extract the knowledge. All other agents that are present in the system and need to access the knowledge base or other data must receive the template from this agent.

Signal processing agent (SPAgent): This is a simple worker agent that has the main knowledge and skills of how to collect and process the given signal. One instance of this type of agent exists for each sensor or type of signal present in the monitoring platform. Because there is no universal method for processing various types of data, this agent receives the information about the signal, the filtering, the sampling rate, and the segmentation and loads the appropriate methods or techniques for that specific signal. This agent does not directly access the knowledge base, and the processed data are stored in a local database. Its presence and the given tasks (which methods to load) are coordinated by the Activity agent and Vital sign agent(s).

Vital sign agent: One instance of this type of agent exists for each monitored vital sign. Its main task is to extract the vital sign measurements from the local database, where the processed data were stored, and to update that knowledge in the KB. This agent receives the information from the Manager agent about the health sensors and the signal type and requests the creation of a signal processing agent. The detailed description of vital sign extraction is presented in Section 3.4.

Activity agent: One instance of this type of agent exists, and its main task is to recognize the user’s physical activity. When this agent is introduced in the system, it receives the location of the database from which the physical activity data are loaded in order to train the activity recognition model. The recognized activity is stored in the KB. Because for activity recognition, data from different wearable sensors, that are present, can be used and fused, this agent requests the creation of, and coordinates, different signal processing agents. The detailed description of activity recognition by this agent is presented in Section 3.5.

Health status agent (HSAgent): This agent has the main scope to evaluate the user’s health status. It uses the vital signs’ database, which contains samples of vital sign measurements from various users performing different physical activities, in order to automatically extract the threshold ranges (intervals) for each monitored vital sign separately. Ranges are marked as a normal range (green zone), altered range (yellow zone), or emergency range (red zone). The extracted ranges are stored in the form of rules in the KB. This agent will write one rule for each range and for every physical activity that is present in the knowledge base. Based on these rules, the user’s health status is evaluated. The detailed description of knowledge extraction by this agent is presented in Section 3.3.

Alert agent: This type of agent has the main task to select the appropriate action based on the patient’s health situation determined by the Health status agent. It communicates to external agents to display warning notifications or alerts for the user or relatives/care providers about the user’s health status. In this version of the architecture, it implements a simple *Alert protocol*: it sends a *warning* message to the User agent when one of the vital signs is in the yellow zone and an *alert* message if two vital signs are in the yellow zone; an *alert* message to the Caretaker agent if one vital sign is in the red zone; and an *alert* message to the Emergency service agent if two vital signs are in the red zone. The content of the messages refers to the name of a vital sign that exceeds the normal range, its value, and the activity that the user was performing.

External agent: This type of agent represents each agent (User agent, Caretaker agent, and Emergency service agent) from the external systems that interact with the architecture, representing different persons or entities in the application that must be informed when the user’s health status worsens. Their main task is to receive and display warning notifications or alerts.

### 3.3. Vital Sign Ranges

To extract the vital sign ranges, we applied Karl Pearson’s theory of histograms. For each physical activity present in the database, the Health status agent extracts the interval (all measurements for one vital sign recorded during that activity) and divides it into five subintervals (bins). Each bin that contains a number of samples higher that the mean number of samples is marked as the green zone. Therefore, the the normal range for that vital sign is extracted as being between the minimum and maximum value of that bin. If the bins marked as the green zone are adjacent, then the normal range is between the minimum value from the first bin and the maximum value of the last bin marked as the green zone. Then, this agent extracts the ranges from the bins that contain fewer samples (the number of samples in the bin is less than mean number of samples), which are marked as the yellow zone. In case there exists a bin, marked as the yellow zone, between two bins marked as the green zone, that bin will also be marked as the green zone. For values lower than the normal range, it checks if the first bin is not marked as the green zone, then the altered range is extracted as being between the minimum value in the interval and the minimum value of negative one of the first bin marked as the green zone. The same proceeds with the physiological measurements that are higher than normal range. It checks if the last bin is not marked as the green zone, then the altered range is extracted as being between the maximum value of positive one of the last bin marked as the green zone and the maximum value in the interval. Finally, the red (emergency) zone is defined as being the value lower than the minimum or higher than the maximum in the extracted interval.

We chose this approach because the Health status agent can adapt the ranges to different target users. Based on their demographics, such as age group, geographical region, or health information, the appropriate user data can be selected from the vital signs’ database to perform range extraction.

### 3.4. Vital Sign Extraction

If in the application, the heart rate and breathing rate are monitored, there exists one agent for each vital sign (BR agent and HR agent). In the absence of a dedicated sensor that can send the raw physical measurement data, these agents implement the possibility to extract or estimate vital signs from the ECG signal. Because both vital sign agents access the same resource, a single signal processing agent is created. This agent adopts the available filtering method and only stores the data in the local database. No segmentation is performed by this agent because the BR agent and HR agent use different window sizes to extract the measurements. In this work, the ECG signal was filtered using the “notch” filter of the second order with a cutoff frequency of 0.05 Hz and a quality factor of 0.005 Hz, as presented in [39]. This filter is useful especially for removing the baseline wander from ECG signals. The filtered signal is stored for further processing by the two vital sign agents. The extraction of the vital signs from the ECG signal was performed using the methods described in [39,40,63].

#### 3.4.1. HR Agent

In order to extract the HR, filtered physiological data are segmented using a non-overlapping sliding window approach. We selected the size of the window as 4 s. To detect the heartbeats, for each given window size, this agent applies a rolling window technique of 0.25 s on both sides of the data points and calculates the rolling average. For each rolling segment in the given window of 4 s, the regions of interest (ROI) where the signal amplitude is larger than the rolling average (if the signal is above the local mean) are marked. The positions of the R peaks are taken as being the highest point in the marked ROI. Then, this agent computes the RR-interval, the distance, between each consecutive detected peak. The distance is converted to *ms*. The positions of the R peaks and RR-interval lists are retained for further processing. The heart rate is determined by the interval between two consecutive R peaks, so the average RR-interval divided by 1 min is computed as being the average *HR* in a given window of 4 s. The computed heart rate measurement is stored in the KB.

(1) $$HR_{w}=\frac{T}{RRI_{mean}}$$

where *T* represents the time of 1 min (6000 ms), $RRI_{mean}$ is the average value of the RR-interval distances, and *w* represents the given signal window.

**Figure 3:**
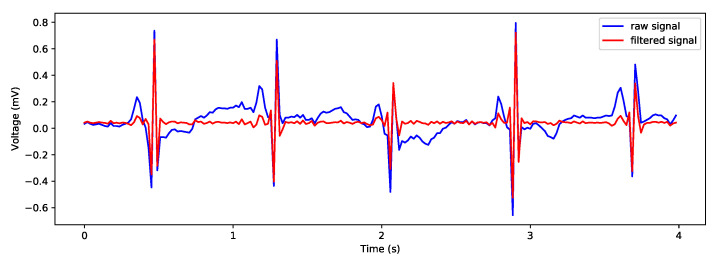
Raw and filtered ECG signal with visible R peaks in a 4 s window.

Because the signal can contain noise, especially produced by moving artifacts, we applied the signal quality index algorithm proposed in [64] for labeling the signal as “bad signals” if the extracted HR was above 190 bpm. The raw ECG signal with visible R peaks, before and after applying the filtering technique, is presented in Figure 3.

#### 3.4.2. BR agent

Because the BR agent uses the heart rate in order to estimate the breathing rate, with the HR agent present in the system, this agent does not need to extract the R peaks from the signal and compute the RR-interval by itself. It subscribes to the HR agent to receive the needed information. If the HR agent is not present, this agent loads the R peak detection method. To estimate the BR, a sliding window of 20 s was selected with an 80% overlap, meaning that this agent extracts the BR every 4 s from the previous 20 s interval. After receiving the RR-intervals (distance between peaks) in *ms* from the HR agent and computing the HR for each interval, a cubic spline interpolation method was performed to obtain the EDR (ECG-derived respiration) waveform. In the case of a low sampling rate of 50 Hz, the data was up-sampled four times. After detecting the respiratory peaks, this agent computed the mean respiratory frequency for the given window size.

(2) $$BR_{w}=\frac{N_{RespPeaks}}{N_{w}}$$

where *w* represents the given window, $N_{RespPeaks}$ is the number of detected respiratory peaks in *w*, and $N_{ResPeaks}$ is the size of the given window.

### 3.5. Activity Recognition

In order to recognize the user’s physical activity, the Activity agent uses only accelerometer data. In case of multiple sensors present in the system that have the accelerometer sensing capability and because each sensor can have a different sampling rate or different communication protocol, the Activity agent requests a signal processing agent that manages each sensor’s data. It informs these agents about the segmentation method that must be performed. The processed data are stored in the local database.

#### 3.5.1. Signal Processing

**Figure 4:**
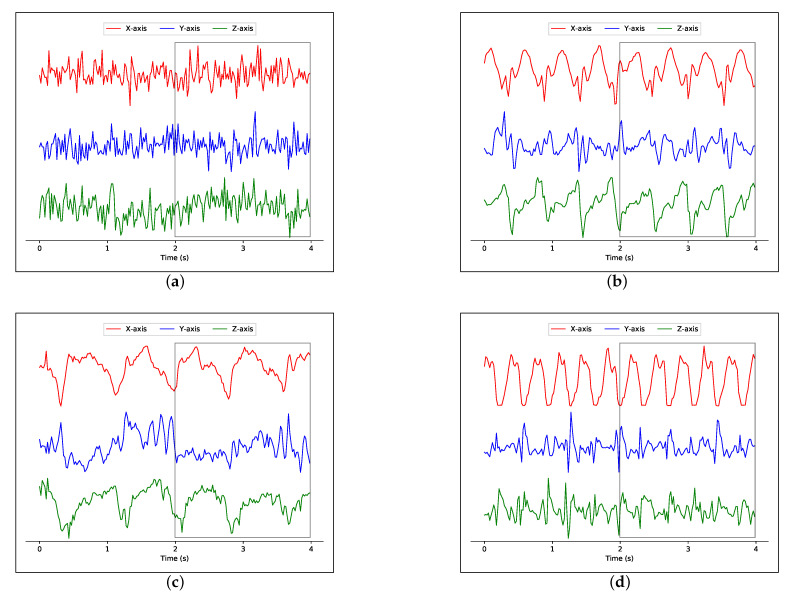
Tri-axial accelerometer data from the IMU placed on the chest for different physical activities (x-axis, top; y-axis, middle; z-axis, bottom): (**a**) standing; (**b**) walking; (**c**) climbing stairs; (**d**) running.

The raw accelerometer signal, containing readings from all three axes (*x, y, z*), is segmented using a fixed-size non-overlapping sliding window (FNSW) of 2 s. We chose a window length of 2 s because it was sufficient to recognize simple physical activities. In Figure 4 is presented the accelerometer signal patterns for different activities of daily living.

Different physical activities may have similar characteristics, thus making it difficult to represent one activity uniquely. In this context, the Activity agent computes different features in order to discriminate between physical activities. From the segmented data, in a local database, a feature vector is extracted for the given window size. The Activity agent can extract features from the original raw signal or compute the signal magnitude (*SM*), from which it extracts the feature vector. The signal magnitude is computed using the following formula:

(3) $$SM\left(i\right)=\sqrt{x_{i}^{2}+y_{i}^{2}+z_{i}^{2}}$$

where *x_i_*, *y_i_*, and *z_i_* are the *i*-th readings of the three axes of the accelerometer’s signal in a given window.

Features from both the time domain and the frequency domain were extracted. The frequency domain was obtained by applying the fast Fourier transform (FFT) on the original signal. In total, six feature vectors were considered for further analysis:

- *xyz_t*—feature vector from the original signal in the time domain;
- *xyz_f*—feature vector from the FFT components of the original signal;
- *xyz_tf*—aggregated features from *xyz_t* and *xyz_f*;
- *mag_t*—feature vector from the computed magnitude of the original signal in the time domain;
- *mag_f*—feature vector from the FFT components of *mag_t*;
- *mag_tf*—aggregated features from *mag_t* and *mag_f*.

For the time domain features, we considered the statistical features such as: mean value (*mean*), standard deviation (*std*), minimum value (*min*), maximum value (*max*), median absolute deviation (*mad*), interquartile range (*iqr*), variance (*var*), zero crossing (*zc*), root mean square (*rms*), skewness value (*s*), and kurtosis value (*k*). Furthermore, we considered the energy measure (*e*) computed as the sum of squares divided by the window length (number of readings):

(4) $$E_{x}=\frac{\sum {{\left|x_{i}\right|}^{2}}^{}}{w}$$

where *x_i_* is the reading in the *x*-axis and *w* represents the window length. This feature has shown good results in discriminating the intensity of physical activities [65].

All of the above-mentioned features were computed and extracted for each of the three axes. Therefore, three attributes of each feature would be present in the feature vector obtained from the original signal, and a single attribute of each feature would be present in the feature vector from the signal’s magnitude. Further, we considered the signal magnitude area (*sma*) as the sum of the integrals of the magnitude of all three accelerator axes. This feature is linearly related to the metabolic energy expenditure of the subject [43].

(5) $$SMA_{\left(x,y,z\right)}=\frac{\sum (\left|x_{i}\right|+\left|y_{i}\right|+\left|z_{i}\right|)}{w}$$

where *x_i_*, *y_i_*, and *z_i_* represent each reading of the three axes of the accelerometer’s signal in a given window and *w* is the window length.

Applying only on the raw signal, where all three signals from the corresponding axes were present, we computed also the correlation among the axes (*xy*, *xz*, and *yz*) [66].

(6) $$Corr_{\left(x,y\right)}=\frac{cov(x,y)}{\delta_{x}\delta_{y}}$$

where *cov(x,y)* is the ratio of the covariance between the *x*-axis and *y*-axis and δxδy is the product of the standard deviations of the two axes.

In total, the feature vector contained 40 and 13 attributes for the raw signal vector and magnitude vector, respectively. For the frequency domain, we extracted all the features computed for the time domain, and in addition, we extracted the signal entropy (*se*) and the index of the maximum frequency component (*im*).

#### 3.5.2. Activity Classification

The recognition of physical activities was based on Waikato Environment for Knowledge Analysis (WEKA) [67,68], an open-source data-mining toolkit and libraries for Java. The parameters used for the classifiers and for selecting the best feature set were the default parameters to ensure reproducibility. The Activity agent uses the WEKA library to train a model using data from the *Activity database* and to classify activities based on the selected model. All six mentioned feature vectors were analyzed, and the model selection, as well as the optimal feature set were based on the highest accuracy score obtained. Most of the standard and state-of-the-art supervised machine learning models, such as support vector machine (SVM), random forest (RF), and k-nearest neighbors (KNN), were used. Furthermore, a multilayer perceptron (MLP) neural network with three hidden layers was implemented. We chose these classifiers because they showed a good performance on similar activity recognition work in the literature. Model evaluation was performed using both the k-fold cross-validation and the leave-one-subject-out evaluation methods.

Further, after selecting the model with the best performance and the feature vector on which that performance was obtained, the Activity agent selects the optimal number of features to improve the classification accuracy. The feature reduction was based on feature importance, and for each iteration, features with the lowest importance were eliminated and the model retrained with the remaining features until the most discriminatory features remained.

### 3.6. Team of Agents

**Figure 5:**
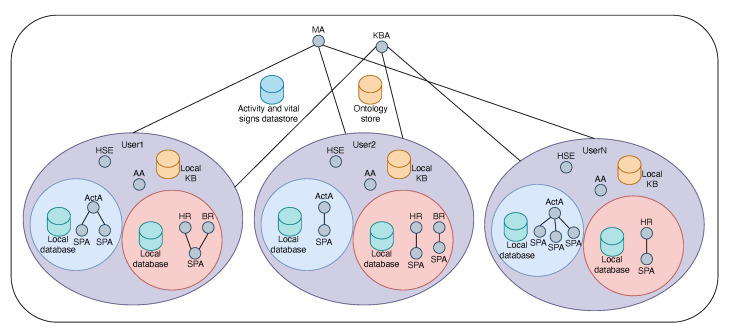
Hierarchical architecture and team clusters for multi-user monitoring.

The proposed architecture can perform with a single user in the environment, addressed mostly toward home monitoring systems, or with multiple users in the environment, addressed toward nursing homes for the elderly or rehabilitation centers. In the case of multiple users present in the monitoring platform, a single instance of the Manager agent and the Knowledge base agent exists, and a team of agents is created for each monitored user. The information is shared only between team members, and each team has its blackboard. The hierarchical architecture is presented in Figure 5.

## 4. Experimental Results and Discussion

In order to validate the proposed multi-agent architecture, we performed different experiments on a real-life dataset that contained both body motion data and physiological data obtained from subjects while performing different activities of daily living. The agents were implemented in the Java Agent Development Environment (JADE) (https://jade.tilab.com/, accessed on 10 January 2021) framework Version 4.5, and we used the Apache Fuseki (https://jena.apache.org/documentation/fuseki2/, accessed on 10 January 2021) server for the triple store. We chose this lightweight triple store server because it can be easily installed locally and can also run on devices with limited computing resources. The triple store was modeled using the presented ontologies. Apache JENA (https://jena.apache.org, accessed on 10 January 2021), an open-source Java framework for building linked data applications, was used for the interaction between agents and the triple store. All the experiments were conducted on a macOS computer equipped with an Intel Quad-Core i7 2.5 GHz CPU and 16 GB 1600 MHz DDR3 RAM.

### 4.1. Dataset

Experiments were performed using the publicly available MHEALTH dataset accessible online at UCI Machine Learning Repository (http://archive.ics.uci.edu/ml/datasets/MHEALTH+Dataset, accessed on 12 May 2021). This dataset, initially introduced and described in [45,69], contains the body motion and physiological measurements from 10 subjects, of diverse profiles, while performing different activities of daily living. Considering the ethical aspects regarding the subjects’ privacy, the dataset is fully neutralized; therefore, explicit identifiable data are absent from the dataset. The data were collected in an out-of-lab environment with no restriction on how the physical activities had to be executed, with the exception that the subjects should try their best when executing them. The collected data were obtained from three Shimmer2™ inertial measurement units (IMUs) placed on the subjects’ chest, right wrist, and left ankle. Data were recorded at a 50 Hz sampling rate for all sensing modalities. In this work, we considered only the triaxial accelerometer data from all three wearable sensors, used by the Activity agent, and the lead Idata from the two-lead ECG obtained from the IMU placed on the subjects’ chest, used for vital sign extraction by the Vital signs agents. From the total of twelve physical activities that were present in the dataset, all eight common activities of daily living, with various MET scores that corresponded to a light, moderate, and vigorous intensity of activities, were taken into consideration. We excluded the remaining four activities, frontal elevation of the arms, crouching, waist bends forward, and jump front and back, because these are more exercise-/fitness-related activities, and also, based on the Compendium of Physical Activities [70], we could not determine the MET score or intensity of these activities. The activities that were considered in this work were: standing, sitting, and lying, which are posture-related activities; walking and climbing stairs, which are motion-related activities; and cycling, jogging, and running, which are more dynamic and sports-related activities. These activities were performed for a 1 min range by each subject. The selected activities and their updated MET score according to [70] are presented in Table 1.

### 4.2. Activity Recognition

#### 4.2.1. Classifiers’ Results

We analyzed all six feature vectors, which were presented in Section 3.5.1, separately in order to evaluate the classifiers’ performance and from which signal the features had to be extracted. In this section, we consider the signals obtained from the IMU on the chest because it provided the best performance, as was reported in related research work. Feature fusion, from different body-attached sensors, will be discussed in Section 4.2.3.

For the model evaluation, we chose the four most present evaluation measures in the literature: accuracy, precision, recall, and F-measure. The accuracy measure was computed by dividing the true positives (TPs, the number of correctly classified instances of one activity) by N (the total number of instances of that activity present in the dataset). The precision measure was computed as a division of the TPs by the sum of TPs and false positive (FPs, the number of instances of other activities that were misclassified as the target activity). The recall measure was computed by dividing the TP by the sum of the TPs and False Negatives (FNs, the number of target activity instances that were classified as other activities). The F-measure was computed as the harmonic mean of precision and recall, dividing the product of the precision and recall by the sum of these two measures.

(7) $$Accuracy=\frac{TP}{N}$$

(8) $$Precision=\frac{TP}{TP+FP}$$

(9) $$Recall=\frac{TP}{TP+FN}$$

(10) $$F\;-\;measure=2\times \frac{Precision\times Recall}{Precision+Recall}$$

**Figure 6:**
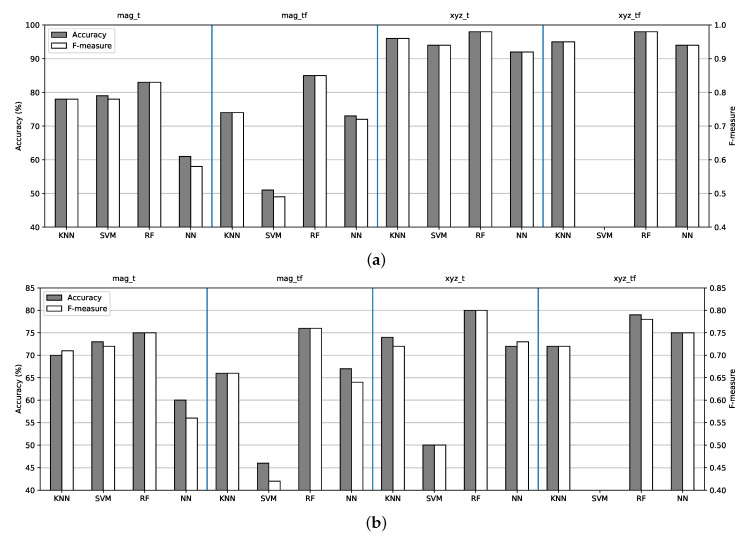
Classifiers performance. (**a**) Ten-fold cross-validation; (**b**) LOSO cross-validation.

The machine learning models (KNN, SVM, RF) were trained using extracted features from the raw signals, while the NN model was trained on raw signals in order to extract features by themselves. Machine learning models extracted all the features presented in Section 3.5.1, after which they selected the most discriminatory feature set. The feature selection method will be discussed in the next section. We used two methods to evaluate the models’ performance, a 10-fold cross-validation method and a leave-one-subject-out (LOSO) cross-validation method. The k-fold method randomly splits data into k − 1 folds, which are used for model training, and the evaluation is performed on the remaining fold. We chose a stratified k-fold because this ensures the same number of classes in the validation set while shuffling the data on each run. Using the LOSO method, each fold is made from all the data from one user, which are used for the validation, while the model is trained using the data from the rest of the users. This method is subjectwise and ensures that the data of the target user are not present in the training process. The results were computed by averaging the results of each fold. All the experiments were run 20 times, and the average values, in terms of accuracy and F-measure, are present in Figure 6.

Because the number of classes in the dataset was perfectly balanced, we analyzed only the accuracy score. The best results were obtained from the RF classifier for both cross-validation methods. For the 10-fold cross-validation method, extracting features from the magnitude vector in the time domain (*mag_t*), the classifier achieved an accuracy of 83% (+/− 1.53 std); extracting features from the magnitude vector in both the time and frequency domains (*mag_tf*), the obtained accuracy was 85% (+/− 1.94 std); extracting features from the raw triaxial signal in time domain (*xyz_t*), an accuracy of 98% (+/− 1.06 std) was achieved; and extracting features from the raw triaxial signal in both the time and frequency domains (*xyz_tf*), the classifier obtained an accuracy of 98% (+/− 0.79 std). On the other hand, using the LOSO cross-validation method, extracting features from the magnitude vector in the time domain (*mag_t*), the classifier achieved an accuracy of 75% (+/− 7.45 std); extracting features from the magnitude vector in both the time and frequency domains (*mag_tf*), an accuracy of 76% (+/− 7.70 std) was achieved; extracting features from the raw triaxial signal in the time domain (*xyz_t*), an accuracy of 80% (+/− 11.30 std) was achieved; and extracting features from the raw triaxial signal in both the time and frequency domains (*xyz_tf*), the classifier obtained an accuracy of 79% (+/− 11.80 std). All classifiers obtained worse accuracy scores from the frequency domain-only vector because most of the activities required a longer window size of data in order to obtain the magnitude spectrum of the signal.

**Figure 7:**
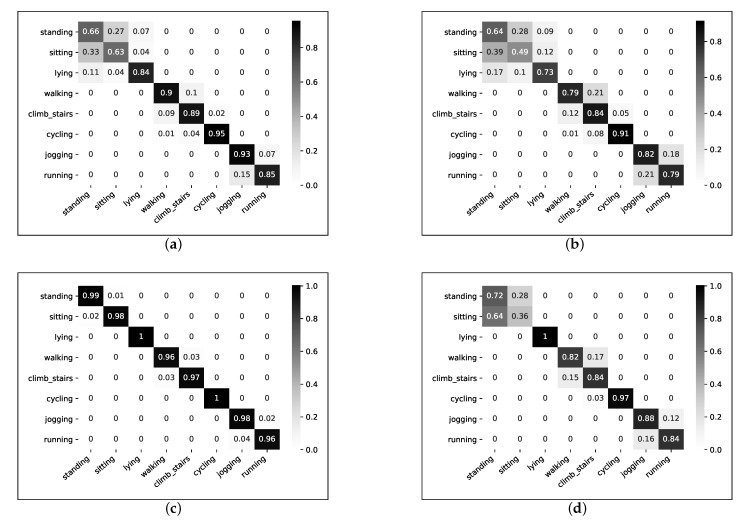
Confusion matrices for the RF classifier on the magnitude and raw accelerometer signals: (**a**) magnitude vector using 10-fold cross-validation; (**b**) magnitude vector using LOSO cross-validation; (**c**) raw triaxial vector using 10-fold cross-validation; (**d**) raw triaxial vector using LOSO cross-validation.

Giving the highest accuracy score, the RF model was selected for activity recognition. Due to the computational cost of the computing frequency domain, and almost the same performance obtained, we analyzed only the RF classifier’s performance in the time domain-only vectors. In Figure 7, we present a confusion matrix for the magnitude and raw signal vectors. As can be noticed, all the misclassifications, for both the raw signal and magnitude vectors, were made among activities with the same intensity. The most confusions were made between the sitting and standing activities and between jogging and running. Better discrimination between activities was obtained by using features from the raw triaxial signal than from the magnitude vector. Therefore, for further analysis, the raw triaxial signal was considered.

Applying the 10-fold cross-validation method, we obtained a very good accuracy of 98%, similar to the related work that used this evaluation method, but this can be seen as a very optimistic evaluation. Considering that this method is subject dependent, it was sufficient for only a small amount of instances from the target subject to exist, and the accuracy increased, thus making it very hard to evaluate new subjects. In real life, a model is developed and trained with a set of people, but the target users are usually unseen by the systems, so it must perform on unseen data. On the other hand, using the LOSO method, a more robust result can be achieved when the system acts on unseen subject data. Using this approach, a cold start problem can be solved when the system must handle the case where there are no data about the target subject. The detailed performance for each activity obtained from the raw triaxial signal using the LOSO cross-validation method is presented in Table 2.

#### 4.2.2. Feature Reduction

**Figure 8:**
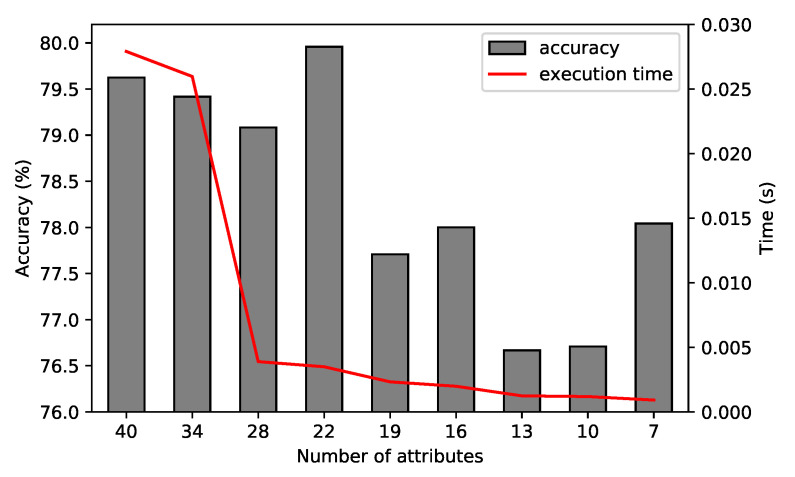
Accuracy and execution time on different feature subsets.

Feature reduction was performed in order to select the best subset of features that discriminated the physical activities. The reduced subset could lead to the improvement of the model’s accuracy and also reduce the computational cost of feature extraction. Initially, the model was trained on all the features discussed in Section 3.5.1. Then, on each iteration, the feature with the least importance was removed from the set, and the model was retrained with the remaining features. On the raw accelerometer data, where signals from all three axes were present, we applied the rule that the same feature for all three axes must remain in the feature set. In that case, the average importance of a triple was compared, not a single attribute. A comparison of the performance of the RF classifier performing on different feature sets and the execution time for each set from the raw triaxial signal originated from the sensor placed on the chest is presented in Figure 8. The execution time reported was obtained by computing the average cost of 20 consecutive runs on each feature subset.

The best accuracy score of 80% was obtained from a feature set that contained 22 attributes. Reducing the number of features more slightly reduced the execution time, but the accuracy significantly decreased. In this work, we did not address the resource constraints. The scalability tests were not the focus of this work, which will be addressed in the future work, so the feature subset was selected based only on the accuracy metrics.

#### 4.2.3. Two-Layer Multimodal Fusion

**Figure 9:**
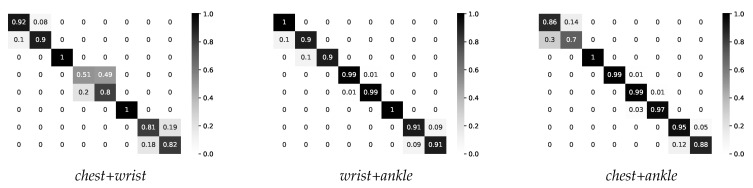
Confusion matrices of feature fusion for all three combinations.

The obtained accuracy of 80% from the chest-worn sensor was not sufficient for a good activity recognition system, so we analyzed different sensor combinations. In this work, we applied the feature-level fusion only, also known as early fusion. The same features were extracted from two sensors to produce a single feature vector. By combining features from the chest and wrist sensors, we obtained an overall accuracy of 85.25%; from the wrist and ankle sensors, an overall accuracy of 94.83% was obtained; and from the chest and ankle sensors, an overall accuracy of 91.83% was obtained, recognizing all eight types of physical activity. Confusion matrices for all three modalities are present in Figure 9.

If we look at the chest and wrist sensor fusion, we can notice an accuracy of 94% among light-intensity activities (standing, sitting, and lying). Combining features from the wrist- and ankle-worn sensors, an accuracy of 99% was achieved for moderate-intensity activities (walking, climbing stairs, and cycling). Combining the chest and ankle sensors, an accuracy of 92% was achieved for vigorous-intensity activities (jogging and running).

**Figure 10:**
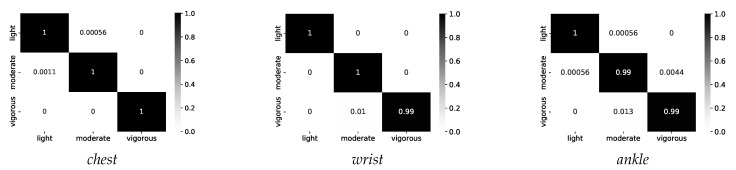
Confusion matrices for detecting the intensity of the activity.

Further, we considered the detection of the intensity of activities, and based on this knowledge, the appropriate sensors were selected for the feature fusion. Therefore, on the first layer of activity recognition, we used a decision tree (DT) classifier based on the J48 model to recognize the intensity of the activity. Almost identical accuracy was obtained from the KNN model, but we chose the DT because of the run-time cost compared to the KNN, which uses more run-time resources. In this step, we reduced the sliding window to 1 s, which was sufficient for a very good classification. The best accuracy was obtained from the magnitude vector with only four features (*mad*, *var*, *rms*, and *iqr*). Furthermore, a very good performance can be obtained from any of the three sensors, as can be seen in Figure 10.

After detecting the intensity, on the second layer, the RF classifier extracted the features from the appropriate combination of sensors and achieved an accuracy of 95.25%. If the intensity of the activity was recognized as light, the features were extracted from the chest and wrist sensors; if the activity was recognized as moderate, the features from the wrist and ankle sensors were extracted; and finally, if the activity had vigorous intensity, the features from the chest and ankle sensors were extracted. The following knowledge was extracted, as presented in Table 3, and stored in the KB.

**Figure 11:**
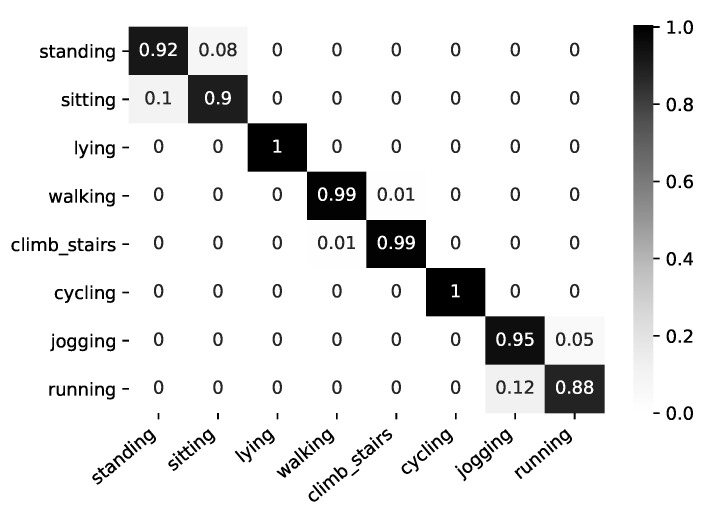
Confusion matrix for the two-layer multimodal fusion approach.

The best performance was obtained by extracting ten attributes from each sensor’s signal. Extracting only seven features slightly improved the accuracy from 92% to 93% for vigorous-intensity activities, while for moderate-intensity activities, the obtained accuracy was the same, but for light-intensity activities, we noticed a drop in performance from 94% to 87%. Therefore, for the final model, we relied on 10 extracted attributes from each sensor. The overall performance of the two-layer multimodal fusion approach is presented in Figure 11. The following features were selected by the system: *mean*, *std*, *sma*, and *rms*.

Applying this technique, we achieved an accuracy of 92% for the standing activity and 90% for the sitting activity, and the rest were misclassified between these two activities. Considering that sitting and standing are both stationary posture activities and the sensors on the chest and wrist can be oriented in the same way during both activities, it can be difficult to distinguish between them using only the accelerometer data and early fusion approach. In the future work, we will also investigate, in case the recognized activity is sitting or standing, if the performance improves by including the data of the sensor placed on the ankle. All instances of the lying activity were classified correctly. For the walking and climbing stairs, we achieved a 99% accuracy, in both cases, the remaining 1% of which was misclassified between these two activities. The cycling activity was very well discriminated, achieving the best accuracy. For the jogging and running activities, we achieved 95% and 88%, respectively, with the remaining misclassified between these two classes. Considering that jogging is defined as “running” slower than 10 km/h, in the different datasets presented in the literature, these two activities are often considered as a single activity. The detailed performance obtained for each activity is presented in Table 4.

In the literature, on the same dataset, using different methods, very good classification results were reported. Using the accelerometer, gyroscope, and magnetometer signals from all three sensors, a temporal sliding window of 5 s, and the leave-one-trial-out with 10-fold cross-validation method, an accuracy of 94.72% was obtained from the ensemble of classifiers in [71]. In [72], using data from all three sensors and sensing modalities, as well as the hold-out method, an accuracy score of 74.85% and 97.15% was achieved for the single base classifier and the hierarchy-based classification method, respectively. In [73], also using data from all three sensors and all three sensing modalities, a 1 s fixed sliding window, and the LOSO cross-validation method, an accuracy of 90.91% was obtained using Adam optimization and the maximum entropy Markov model. With the convolutional neural network (CNN) approach with a sliding window with a size of 60 samples with 50% overlap over the time domain, using the hold-out method and the data from all sensors and modalities, an accuracy of 98.30% was reported in [74]. Furthermore, considering the accelerometer data from all three sensors and the gyroscope data from the wrist and ankle sensors, using the LOSO cross-validation method, an accuracy of 91.94% was obtained in [75]. The long short-term memory-convolutional neural network (LSTM-CNN) model presented in [76] achieved an accuracy of 95.56% using the hold-out method and fixed-width sliding windows of 128 readings, from all three sensors and sensing modalities. A dual-stream recurrent convolutional attention model presented in [77], using the LOSO cross-validation method and data from all three sensors and sensing modalities, obtained an accuracy of 94.0%. In [78], using accelerometer data from the combination of all sensors was investigated. The best accuracy of 91.64% was obtained from the fusion of the ankle and wrist sensors, using a 2 s sliding window and the LOSO cross-validation method to estimate the performance of the recognition model.

The proposed activities’ recognition model, using the two-layer multimodal fusion approach, addressing the cold start problem, acting on unseen subject data by applying the subject-independent evaluation method, reducing the number of data transmissions from the sensors, automatically selecting the appropriate combination of body-attached sensors, performed as well as many systems presented in the literature, obtaining a similar accuracy score.

### 4.3. Activity-Aware Vital Sign Thresholds

**Figure 12:**
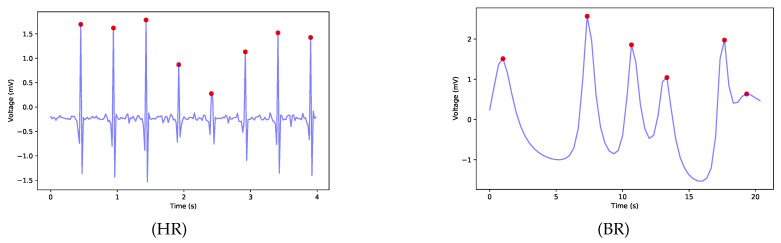
Extracted vital signs, HR and BR, from the ECG signal.

In order to extract the vital signs, HR and BR, we used the lead IECG signal from the dataset. Vital signs were extracted using the methods presented in Section 3.4. For the HR, we used a 4 s non-overlapping sliding window, and for the BR, we used a 20 s window, applying 80% overlap. Therefore, we extracted the BR every 4 s for the previous 20 s interval. In Figure 12, the detected peaks in the ECG signal for the HR obtained from Subject 1 while climbing stairs, with an average HR of 83 bpm and the estimated BR, with an average breathing frequency of 0.30 Hz or 18 brpm, obtained from the same subject while performing a walking activity, are presented. The extracted vital signs were rounded to the nearest integer and stored in the KB. From the dataset, due to the noise caused by moving artifacts, only 88% of the vital signs’ data were obtained, and the remaining were labeled as “bad signal” by the HR agent. In the future work, adaptive filtering methods must be considered.

**Figure 13:**
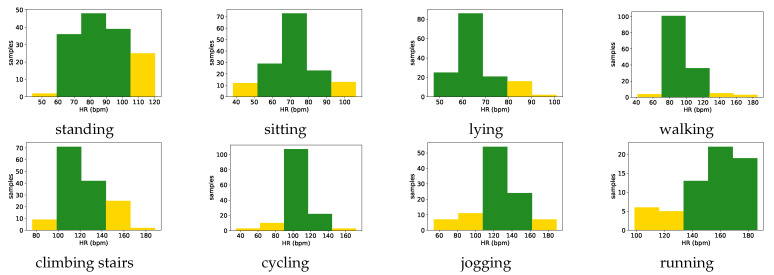
HR threshold ranges.

**Figure 14:**
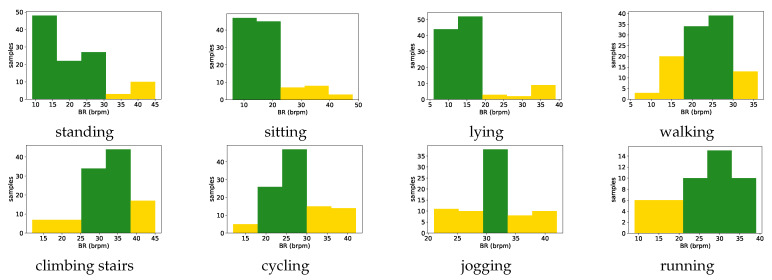
BR threshold ranges.

For each monitored vital sign, the Health agent extracted the new knowledge from the vital signs’ database (dataset). It established the threshold ranges for each vital sign for every activity present in the dataset, as presented in Section 3.3. In the case of the HR ranges only, considering the acceptable error rate of +− 3 bpm in most of the commercial monitors, the extracted intervals were adjusted to the nearest multiple of five. Established ranges, by analyzing the data of all 10 subjects from the dataset, are presented in Figure 13 and Figure 14 for the two monitored vital signs, HR and BR, respectively. The examples of the knowledge in the form of rules, which was extracted from the dataset, are presented in Table 5.

### 4.4. Health Status Monitoring

**Figure 15:**
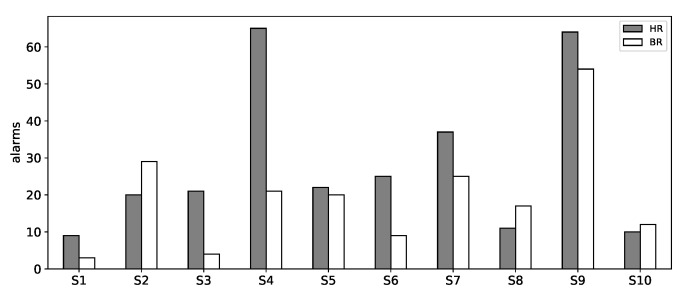
Warnings/alerts generated when physiological measurements exceeded the normal range.

After the independent evaluation of each of the two main components of the proposed architecture (vital sign range extraction and activity recognition), we performed a simulation on each subject from the dataset, acting as the target user, in order to analyze the warnings or alerts that were generated. The purpose of this simulation was strictly to investigate how the system behaved in real-life scenarios. For each subject, the corresponding team was created, and physical activity model training and vital sign threshold range extraction were performed from the data of the other nine subjects from the dataset, except the target user (subject) monitored by the corresponding team. Because only the server-side part of the application was implemented, the external agents, which received the warning/alert messages, ran on the same machine. In addition to this, for each sample from the dataset (for each user’s reading in our simulation), a new *Reading* individual in each local KB was created. This information was used for analysis purposes and was not used by the multi-agent system. The extracted ranges that were used for the vital sign evaluation of each of the ten subjects are presented in Table 6. Because of the small size of the dataset, changing the subjects from which the vital sign intervals were extracted affected some activities’ ranges. For some activities, the monitored subjects had different ranges. A comparison of the warnings/alerts generated (messages sent to the external agents) by the proposed method, for the ten evaluated subjects, is presented in Figure 15.

In the case of the HR, the number of generated warnings/alerts was low for Subjects 1, 8, and 10, generating 9, 11, and 10 warnings/alerts, respectively, meaning that approximately 92% of the samples were classified in the green zone. Between 20 and 25 warnings or alerts were generated for Subjects 2, 3, 5, and 6, resulting in approximately 82% of the samples being classified in the green zone. For Subject 7, there were 37 warnings/alerts generated, resulting in 70% of the samples being classified in the green zone. The number of generated warnings/alerts was very high for Subjects 4 and 9, generating 65 and 64 warnings/alerts, respectively, meaning that approximately 54% of the samples exceeded the green zone. From the dataset, considering all ten experiments, on average, there 77% of the samples classified in the green zone.

In the case of BR, for Subjects 1, 3, and 6, there were 3, 4, and 9 warnings/alerts generated, respectively, resulting in approximately 96% of samples being classified in the green zone. For Subject 8 and Subject 10, there were 12 and 17 warnings/alerts generated, meaning that approximately 88% of the samples fit in the green zone. Between 20 and 29 warnings/alerts were generated for Subjects 2, 4, 5, and 7, resulting in approximately 81% of the samples being classified in the green zone. Most warnings/alerts were generated for Subject 9, 54 in this case, resulting in that only 55% of the samples were classified in the green zone. On average, there 84% of the samples classified in the green zone.

The fewest warnings were generated for Subject 1. Most of the warnings were caused by the lower HR during the jogging and running activities. In both cases, the lower values were recorded at the beginning of these activities.

In the case of Subject 2, most of the warnings/alarms were generated because of the higher HR during the standing activity performed by this user. The extracted HR for this activity was between 81 and 104 bpm. Regarding the BR, warnings/alarms were generated because higher values were recorded during the standing and cycling activities. The user recorded 18–30 brpm and 33–42 brpm, respectively, while performing these two activities.

For Subject 3, the warnings were caused by the recorded HR of 72 bpm during lying, 146–150 bpm during climbing stairs, as well as by higher values, above 145 bpm, recorded during the jogging activity.

Warnings were generated for Subject 4 because the extracted HR was in many cases above the threshold ranges for all light- and moderate-intensity activities. Only three warnings were recorded for high-intensity activities. Regarding the BR, warning alarms were generated because of the lower BR during the running activity for which the extracted values ranged between 9 and 33 brpm.

For Subject 5, warnings/alerts were generated because of the higher HR during walking and climbing stairs. The recorded values were 77–176 bpm and 107–176 bpm while performing the walking and climbing stairs activity, respectively. Regarding the BR, warnings/alarms were generated because of the lower BR during climbing stairs for which 12–24 brpm was recorded for the subject.

In the case of Subject 6, the automatically extracted HR threshold range for the standing activity was 75–105 bpm, which contained the highest value for the lower limit, compared to the threshold ranges of the other subjects, therefore resulting in a warning for each sample because the recorded values from this subject were all below that range.

For Subject 7, most of the warnings/alarms were caused because of the higher HR during the standing and sitting activities performed by this user. The extracted HR values during these activities ranged between 74 and 110 bpm. The computed BR for this subject was very high, in most of the cases exceeding the threshold ranges during the cycling and jogging activities.

In the case of Subject 8, only a few samples fit below the threshold ranges, for the obtained HR, during different activities. Regarding the BR, most of the warnings/alerts were generated because the extracted values were below the threshold range for the walking activity.

For Subject9, the highest number of warnings/alerts was generated. Regarding the HR, almost all samples from the standing, ranging between 61 and 66 bpm, and running, ranging between 98–162 bpm, activities exceeded the threshold ranges. Furthermore, the BR, of 30–42 brpm, was higher than the threshold ranges for almost all samples from the standing, sitting, and lying activities. The BR was the highest among the monitored subjects, during these activities, and was possibly caused by noise in the signal. This issue, by applying different filtering techniques, will be investigated in the future work.

For Subject 10, a few warnings/alerts were generated for the HR during different activities. Most of them (three) were caused by the lower HR during running, recorded from the sample from the beginning of the activity. Regarding the BR, warnings/alerts were generated because of the lower values recorded during the jogging and running activities.

Regarding the number of alerts (in the case that both of the vital signs were out of the green zone), none were generated for Subjects 1, 6, and 10; for Subject 3 and Subject 8, there were three and two alerts registered, respectively (approximately 2% of the total monitored samples from these subjects); for Subject 4 and Subject 7, only six were registered from each subject (5% of the total monitored samples from these subjects); eight were registered for Subject 2 (7% of total monitored samples from this subject); fourteen for Subject 5 (12% of total monitored samples from this subject); and twenty-nine for Subject 9 (24% of total monitored samples from this subject).

Regarding the alerts received by the Caretaker agent (when at least one of the physiological measurements exceeded the yellow zone), only the cases of Subject 5, Subject 7, and Subject 9 were registered. For Subject5, from a total of nine alerts, four alerts were received because of the high HR, which exceeded the above range of 150 bpm of the yellow zone for climbing stairs and five alerts in the case of the walking activity because the measurements exceeded the above range of 135 bpm of the yellow zone for that activity. A single alert was registered for Subject 7 because of the elevated HR, of 106 bpm, during the sitting activity (exceeding the above range of 105 bpm of the yellow zone). In the case of Subject 9, from a total of 22 alerts, one was registered because of the HR of 54 bpm during jogging, exceeding the bottom range of 90 bpm, and three were registered for the lower HR, of 58–69 bpm, which exceeded the bottom range of 120 bpm for the running activity, while eighteen were registered because of the very high BR, of 38–48 brpm, during the standing (6 alerts), sitting (3 alerts), and lying (9 alerts) activities.

Because there was no demographic information present in the dataset, in this work, we selected the data of all nine subjects, which were used for each target user. Considering that subjects had diverse profiles, the method showed promising results. However, in order to reduce the number of warnings/alerts, a larger dataset must be considered, including a larger number of subjects in different categories, in order to select only the data from the subjects with similar characteristics, because users with different characteristics, referring to age or health status, may perform physical activities differently. As in the case of the cycling activity, it can be performed with different intensities, as well as in the case of climbing stairs. For generating warnings/alerts also, the previous activity must be considered because when performing an activity with a higher intensity prior to light activities, the vital sign measurements would remain elevated.

## 5. Conclusions and Future Work

In this work, we proposed a multi-agent architecture for activity-aware vital sign monitoring. The architecture included several predefined types/roles of agents. Each role had a set of tasks that were executed in order to process the signal from the sensors, monitor the vital signs, recognize the user’s physical activity, and alert different entities when the user’s vital signs exceeded the normal ranges. Because the vital signs were related to the physical activity performed by the user, these ranges were extracted automatically for each physical activity known by the system. To better suit the monitored user, these ranges must be obtained automatically from other users with similar characteristics as the monitored user, thus eliminating the manual threshold ranges’ configuration either by the end user or by the medical personnel.

Because activity recognition was the main component in our architecture and vital sign ranges depend on the recognized activity, we dedicated much work in this direction. The system recognized eight simple activities of daily living from the accelerometer data of different body-attached sensors. Features were extracted from both the time and frequency domain, either from raw signals or from the computed magnitude of the signal, for analysis and the best approach selected. The system selected the most discriminant features based on their importance. The system used a two-layer multimodal fusion, in order to fuse data from different available sensors: on the first layer, the intensity of the activity is considered, which can be obtained from each sensor using the decision tree classifier, and based on the intensity, on the second layer, data were fused from those sensors that gave the best accuracy for activities with that intensity.

The system was validated on a real-life dataset, which contained activity data from three body-attached sensors, and using the random forest classifier, overall, the best accuracy obtained from the system was 95.25% by applying the intersubject cross-validation method. Assuming that in real-life, a user performs one activity for some period of time and does not change the type of activity performed very often, by using the two-layer fusion model, the remaining sensor that is not used for recognizing that activity can be put in sleep mode. In the case of the malfunction of one sensor, worst accuracy by using only the two remaining sensors was 84.50%. To cope with the data heterogeneity, the system loaded different domain-specific ontologies. Individuals from these ontologies were used to model data in the knowledge base.

The proposed architecture is flexible and easily extensible to a different context, so it can be implemented either for monitoring a single user in home settings or to monitor multiple users in nursing homes for the elderly or in rehabilitation centers.

Considering the noise that is produced by moving artifacts, when subjects performed the jogging and running activity, the vital signs were extracted from only 88% of the dataset. For the future work, we intend to implement adaptive filtering methods for physiological signals based on the recognized activity.

We intend to extend the architecture with different specialized or expert agents to be able to recognize different heart anomalies, for example, an agent that detects atrial fibrillation or other irregular heartbeats, as well as agents that can extract other vital signs.

One of the drawbacks of the system was the size of the dataset used because the system performance relied on the given data. Using a larger dataset, preferably with users from different age categories and/or with different health conditions, could give a better image in terms of activity classification, because younger people and elderly people can perform the same activity in different modes, especially for vital sign extraction, because the vital sign ranges related to the activities may be different in different age groups and even more if the individuals have different health conditions.

Because the scalability test was not the main focus of this research, in the future, we intend to address this issue regarding the execution time for the feature extraction, the feature subset selection, as well as the machine learning model training and selection.

## Figures and Tables

**Table 1:** Physical activities and MET scores.

Physical Activity	MET
*Light-intensity activities*	*<3*
standing	1.3
sitting	1.3
lying down	1.0
*Moderate-intensity activities*	*3–6*
walking	3.5
climbing stairs	4.0
cycling	4.0
*Vigorous-intensity activities*	*>6*
jogging	7.0
running	8.0

**Table 2:** Activities’ recognition rate for the RF classifier using the LOSO cross-validation method.

Physical Activity	Accuracy	Precision	Recall
standing	0.72	0.53	0.72
sitting	0.36	0.56	0.36
lying	1.0	1.0	1.0
walking	0.82	0.84	0.82
climbing stairs	0.84	0.81	0.84
cycling	0.97	0.99	0.97
jogging	0.88	0.85	0.88
running	0.84	0.87	0.84

**Table 3:** Rules’ extraction for the intensity of activity.

IF intensity=light THEN fuse chest sensor AND wrist sensor
IF intensity=moderate THEN fuse wrist sensor AND ankle sensor
IF intensity=vigorous THEN fuse chest sensor AND ankle sensor

**Table 4:** Activities’ recognition rate using the two-layer multimodal fusion approach.

Physical Activity	Accuracy	Precision	Recall
standing	0.92	0.90	0.92
sitting	0.90	0.92	0.90
lying	1.0	1.0	1.0
walking	0.99	0.99	0.99
climbing stairs	0.99	0.99	0.98
cycling	1.0	1.0	1.0
jogging	0.95	0.89	0.95
running	0.88	0.94	0.88

**Table 5:** Rules’ extraction for vital signs threshold ranges.

IF activity=walking THEN normal HR range is 70–125 bpm
IF activity=running THEN altered HR range is 100–135 bpm
IF activity=cycling THEN normal BR range is 18–30 brpm
IF activity=sitting THEN emergency BR range is <8 brpm

**Table 6:** Vital signs’ green zone ranges during physical activities for each monitored subject.

Physical Activity	Subject 1		Subject 2		Subject 3		Subject 4		Subject 5	
	HR	BR	HR	BR	HR	BR	HR	BR	HR	BR
standing	60–105	9–30	60–90	9–23	60–105	9–30	60–100	9–30	60–110	9–30
sitting	55–95	6–22	55–80	8–22	55–80	6–22	55–80	6–22	55–80	6–22
lying	45–90	6–18	55–80	6–18	45–70	6–18	45–70	6–18	45–70	6–18
walking	70–125	12–30	65–125	12–30	70–125	12–30	70–100	12–30	85–110	18–30
climbing stairs	100–160	25–38	100–145	25–38	100–145	24–40	100–145	25–38	105–125	28–37
cycling	60–120	18–30	60–125	16–28	90–145	18–30	90–145	18–30	75–120	18–27
jogging	105–160	29–33	105–160	29–33	105–145	28–33	110–160	29–33	110–160	28–33
running	150–185	21–38	150–185	20–37	150–185	21–38	150–185	23–34	150–185	21–38
**Physical Activity**	**Subject 6**		**Subject 7**		**Subject 8**		**Subject 9**		**Subject 10**	
	**HR**	**BR**	**HR**	**BR**	**HR**	**BR**	**HR**	**BR**	**HR**	**BR**
standing	75–105	9–30	60–105	9–30	60–105	9–30	65–105	9–28	60–105	9–30
sitting	55–80	6–22	60–85	6–22	55–80	6–22	60–85	6–18	55–95	6–22
lying	45–70	6–18	45–70	6–18	45–70	6–18	45–70	12–18	45–90	6–18
walking	70–125	18–30	70–125	12–28	70–125	18–30	70–115	14–30	70–125	12–30
climbing stairs	100–145	25–38	100–145	25–38	100–145	25–38	110–145	25–38	100–145	25–38
cycling	90–145	18–30	90–145	18–30	90–145	18–30	80–135	22–30	90–145	18–30
jogging	110–160	28–33	110–160	28–33	110–160	28–33	95–150	28–33	110–160	28–33
running	150–185	21–38	150–185	21–38	150–185	21–38	155–185	21–38	150–185	21–38

## Data Availability

The MHEALTH dataset, introduced and described in [45,69], is available online at http://archive.ics.uci.edu/ml/datasets/MHEALTH+Dataset (accessed on 12 May 2021).

## References

[1] World Health Organization (WHO) (2014). Global Status Report on Noncommunicable Diseases 2014.

[2] Tanaka T., Fujita T., Sonoda K., Nii M., Kanda K., Maenaka K., Okochi S., Higuchi K., Alex Chan Chun Kit Wearable health monitoring system by using fuzzy logic heart-rate extraction. Proceedings of the World Automation Congress 2012.

[3] Lee D.H., Rabbi A., Choi J., Fazel-Rezai R. (2012). Development of a Mobile Phone Based e-Health Monitoring Application. Int. J. Adv. Comput. Sci. Appl..

[4] Kannan S. (2012). Wheats: A Wearable Personal Healthcare and Emergency Alert and Tracking System. Eur. J. Sci. Res..

[5] Watanabe T., Saito H. Tests of wireless wearable sensor system in joint angle measurement of lower limbs. Proceedings of the 2011 Annual International Conference of the IEEE Engineering in Medicine and Biology Society.

[6] Felisberto F., Costa N., Fdez-Riverola F., Pereira A. (2012). Unobstructive Body Area Networks (BAN) for Efficient Movement Monitoring. Sensors.

[7] Anania G., Tognetti A., Carbonaro N., Tesconi M., Cutolo F., Zupone G., Rossi D.D. Development of a novel algorithm for human fall detection using wearable sensors. Proceedings of the 2008 IEEE Sensors.

[8] Baek W.S., Kim D.M., Bashir F., Pyun J.Y. Real life applicable fall detection system based on wireless body area network. Proceedings of the 2013 IEEE 10th Consumer Communications and Networking Conference (CCNC).

[9] Felisberto F., Fdez.-Riverola F., Pereira A. (2014). A Ubiquitous and Low-Cost Solution for Movement Monitoring and Accident Detection Based on Sensor Fusion. Sensors.

[10] Van Dam K., Pitchers S., Barnard M. Body area networks: Towards a wearable future. Proceedings of the Wireless World Research Forum (WWRF) Kick off Meeting.

[11] Subbe C.P., Kruger M., Rutherford P., Gemmel L. (2001). Validation of a modified Early Warning Score in medical admissions. Qjm.

[12] Hart J. (2015). Normal resting pulse rate ranges. J. Nurs. Educ. Pract..

[13] Nealen P.M. (2016). Exercise and lifestyle predictors of resting heart rate in healthy young adults. J. Hum. Sport Exerc..

[14] Whyte G., Sharma S. (2010). Practical ECG for Exercise Science and Sports Medicine.

[15] Physical Activity Guidelines Advisory Committee (2008). Physical Activity Guidelines Advisory Committee report 2008: To the Secretary of Health and Human Services. Nutr. Rev..

[16] Riebe D., Ehrman J.K., Liguori G., Magal M. (2017). ACSM’s Guidelines for Exercise Testing and Prescription.

[17] Barrett K. (2012). Ganong’s Review of Medical Physiology.

[18] Loring S.H., Mead J., Waggener T.B. (1990). Determinants of breathing frequency during walking. Respir. Physiol..

[19] Raßler B., Kohl J. (1996). Coordination of Breathing and Walking at Different Treadmill Speed and Slope Levels and its Effects on Respiratory Rate and Minute Ventilation. The Physiology and Pathophysiology of Exercise Tolerance.

[20] Nicolò A., Massaroni C., Passfield L. (2017). Respiratory Frequency during Exercise: The Neglected Physiological Measure. Front. Physiol..

[21] Mcllroy M.B. (1963). The respiratory response to exercise. Pediatrics.

[22] Liu G.Z., Guo Y.W., Zhu Q.S., Huang B.Y., Wang L. (2011). Estimation of Respiration Rate from Three-Dimensional Acceleration Data Based on Body Sensor Network. Telemed. E-Health.

[23] Jetté M., Sidney K., Blümchen G. (1990). Metabolic equivalents (METS) in exercise testing, exercise prescription, and evaluation of functional capacity. Clin. Cardiol..

[24] Micucci D., Mobilio M., Napoletano P. (2017). UniMiB SHAR: A Dataset for Human Activity Recognition Using Acceleration Data from Smartphones. Appl. Sci..

[25] Paul P., George T. An effective approach for human activity recognition on smartphone. In Proceeding of the 2015 IEEE International Conference on Engineering and Technology (ICETECH).

[26] Banos O., Damas M., Pomares H., Rojas I. (2012). On the Use of Sensor Fusion to Reduce the Impact of Rotational and Additive Noise in Human Activity Recognition. Sensors.

[27] Reiss A., Stricker D. (2012). Creating and benchmarking a new dataset for physical activity monitoring. Proceedings of the 5th International Conference on PErvasive Technologies Related to Assistive Environments—PETRA12.

[28] Cottone P., Re G.L., Maida G., Morana M. Motion sensors for activity recognition in an ambient-intelligence scenario. Proceedings of the 2013 IEEE International Conference on Pervasive Computing and Communications Workshops (PERCOM Workshops).

[29] Htike K.K., Khalifa O.O., Ramli H.A.M., Abushariah M.A.M. Human activity recognition for video surveillance using sequences of postures. Proceedings of the The Third International Conference on e-Technologies and Networks for Development (ICeND2014).

[30] Pires I.M., Garcia N.M., Pombo N., Flórez-Revuelta F., Spinsante S., Teixeira M.C. (2018). Identification of activities of daily living through data fusion on motion and magnetic sensors embedded on mobile devices. Pervasive Mob. Comput..

[31] Cleland I., Kikhia B., Nugent C., Boytsov A., Hallberg J., Synnes K., McClean S., Finlay D. (2013). Optimal Placement of Accelerometers for the Detection of Everyday Activities. Sensors.

[32] Sztyler T., Stuckenschmidt H. On-body localization of wearable devices: An investigation of position-aware activity recognition. Proceedings of the 2016 IEEE International Conference on Pervasive Computing and Communications (PerCom).

[33] Ordóñez F., Roggen D. (2016). Deep Convolutional and LSTM Recurrent Neural Networks for Multimodal Wearable Activity Recognition. Sensors.

[34] Chung S., Lim J., Noh K.J., Kim G., Jeong H. (2019). Sensor Data Acquisition and Multimodal Sensor Fusion for Human Activity Recognition Using Deep Learning. Sensors.

[35] Khan R.A., Pathan A.S.K. (2018). The state-of-the-art wireless body area sensor networks: A survey. Int. J. Distrib. Sens. Netw..

[36] Hasan K., Biswas K., Ahmed K., Nafi N.S., Islam M.S. (2019). A comprehensive review of wireless body area network. J. Netw. Comput. Appl..

[37] Mahdiani S., Jeyhani V., Peltokangas M., Vehkaoja A. Is 50 Hz high enough ECG sampling frequency for accurate HRV analysis?. Proceedings of the 2015 37th Annual International Conference of the IEEE Engineering in Medicine and Biology Society (EMBC).

[38] Kwon O., Jeong J., Kim H.B., Kwon I.H., Park S.Y., Kim J.E., Choi Y. (2018). Electrocardiogram Sampling Frequency Range Acceptable for Heart Rate Variability Analysis. Healthc. Inform. Res..

[39] van Gent P., Farah H., van Nes N., van Arem B. (2019). HeartPy: A novel heart rate algorithm for the analysis of noisy signals. Transp. Res. Part F Traffic Psychol. Behav..

[40] Sarkar S., Bhattacherjee S., Pal S. Extraction of respiration signal from ECG for respiratory rate estimation. Proceedings of the Michael Faraday IET International Summit 2015.

[41] Bersch S., Azzi D., Khusainov R., Achumba I., Ries J. (2014). Sensor Data Acquisition and Processing Parameters for Human Activity Classification. Sensors.

[42] Ni Q., Patterson T., Cleland I., Nugent C. (2016). Dynamic detection of window starting positions and its implementation within an activity recognition framework. J. Biomed. Inform..

[43] Khusainov R., Azzi D., Achumba I., Bersch S. (2013). Real-Time Human Ambulation, Activity, and Physiological Monitoring: Taxonomy of Issues, Techniques, Applications, Challenges and Limitations. Sensors.

[44] Lima W.S., Souto E., El-Khatib K., Jalali R., Gama J. (2019). Human Activity Recognition Using Inertial Sensors in a Smartphone: An Overview. Sensors.

[45] Banos O., Villalonga C., Garcia R., Saez A., Damas M., Holgado-Terriza J.A., Lee S., Pomares H., Rojas I. (2015). Design, implementation and validation of a novel open framework for agile development of mobile health applications. BioMed. Eng. OnLine.

[46] Preece S.J., Goulermas J.Y., Kenney L.P.J., Howard D. (2009). A Comparison of Feature Extraction Methods for the Classification of Dynamic Activities From Accelerometer Data. IEEE Trans. Biomed. Eng..

[47] Zheng X., Wang M., Ordieres-Meré J. (2018). Comparison of data preprocessing approaches for applying deep learning to human activity recognition in the context of industry 4.0. Sensors.

[48] Castro D., Coral W., Rodriguez C., Cabra J., Colorado J. (2017). Wearable-based human activity recognition using an iot approach. J. Sens. Actuator Netw..

[49] Uddin M.T., Billah M.M., Hossain M.F. Random forests based recognition of human activities and postural transitions on smartphone. Proceedings of the 2016 5th International Conference on Informatics, Electronics and Vision (ICIEV).

[50] Shakshuki E., Reid M. (2015). Multi-Agent System Applications in Healthcare: Current Technology and Future Roadmap. Procedia Comput. Sci..

[51] Su C.J. (2008). Mobile multi-agent based, distributed information platform (MADIP) for wide-area e-health monitoring. Comput. Ind..

[52] Fuentes-Fernández R., Guijarro M., Pajares G. (2009). A Multi-Agent System Architecture for Sensor Networks. Sensors.

[53] Villarrubia G., Bajo J., Paz J.D., Corchado J. (2014). Monitoring and Detection Platform to Prevent Anomalous Situations in Home Care. Sensors.

[54] Ahmid M., Kazar O., Benharzallah S., Kahloul L., Merizig A. An Intelligent and Secure Health Monitoring System Based on Agent. Proceedings of the 2020 IEEE International Conference on Informatics, IoT, and Enabling Technologies (ICIoT).

[55] Armentia A., Gangoiti U., Priego R., Estévez E., Marcos M. (2015). Flexibility Support for Homecare Applications Based on Models and Multi-Agent Technology. Sensors.

[56] Strang T., Linnhoff-Popien C. A Context Modeling Survey. Proceedings of the First International Workshop on Advanced Context Modelling, Reasoning And Management at UbiComp 2004.

[57] Goldfain A., Smith B., Arabandi S., Brochhausen M., Hogan W. Vital Sign Ontology. Proceedings of the 14th Annual Bio-Ontologies Meeting.

[58] Kim H., Mentzer J., Taira R. (2019). Developing a Physical Activity Ontology to Support the Interoperability of Physical Activity Data. J. Med Internet Res..

[59] Compton M., Barnaghi P., Bermudez L., García-Castro R., Corcho O., Cox S., Graybeal J., Hauswirth M., Henson C., Herzog A. (2012). The SSN ontology of the W3C semantic sensor network incubator group. J. Web Semant..

[60] Villalonga C., Pomares H., Rojas I., Banos O. (2017). MIMU-Wear: Ontology-based sensor selection for real-world wearable activity recognition. Neurocomputing.

[61] Rhayem A., Mhiri M.B.A., Gargouri F. HealthIoT Ontology for Data Semantic Representation and Interpretation Obtained from Medical Connected Objects. Proceedings of the 2017 IEEE/ACS 14th International Conference on Computer Systems and Applications (AICCSA).

[62] Ivascu T. An Energy Efficient Intelligent Wireless Body Area Network for Real-Time Vital Signs Monitoring. Proceedings of the 2015 17th International Symposium on Symbolic and Numeric Algorithms for Scientific Computing (SYNASC).

[63] Van Gent P., Farah H., Nes N., van Arem B. Heart rate analysis for human factors: Development and validation of an open source toolkit for noisy naturalistic heart rate data. Proceedings of the 6th HUMANIST Conference.

[64] Orphanidou C., Bonnici T., Charlton P., Clifton D., Vallance D., Tarassenko L. (2014). Signal Quality Indices for the Electrocardiogram and Photoplethysmogram: Derivation and Applications to Wireless Monitoring. IEEE J. Biomed. Health Inform..

[65] Sugimoto A., Hara Y., Findley T., Yoncmoto K. (1997). A useful method for measuring daily physical activity by a three-direction monitor. Scand. J. Rehabil. Med..

[66] Ravi N., Dandekar N., Mysore P., Littman M.L. (2005). Activity recognition from accelerometer data. Aaai.

[67] Hall M., Frank E., Holmes G., Pfahringer B., Reutemann P., Witten I.H. (2009). The WEKA data mining software. ACM SIGKDD Explor. Newsl..

[68] Witten I., Frank E., Hall M.A., Pal C.J. (2017). Data Mining: Practical Machine Learning Tools and Techniques.

[69] Banos O., Garcia R., Holgado-Terriza J.A., Damas M., Pomares H., Rojas I., Saez A., Villalonga C. (2014). mHealthDroid: A Novel Framework for Agile Development of Mobile Health Applications. Ambient Assisted Living and Daily Activities.

[70] Ainsworth B.E., Haskell W.L., Herrmann S.D., Meckes N., Bassett D.R., Tudor-Locke C., Greer J.L., Vezina J., Whitt-Glover M.C., Leon A.S. (2011). 2011 Compendium of Physical Activities: A second update of codes and MET values. Med. Sci. Sport. Exerc..

[71] Nguyen H.D., Tran K.P., Zeng X., Koehl L., Tartare G. Wearable Sensor Data Based Human Activity Recognition using Machine Learning: A new approach. Proceedings of the ISSAT International Conference on Data Science in Business, Finance and Industry.

[72] Khowaja S.A., Yahya B.N., Lee S.L. (2017). Hierarchical classification method based on selective learning of slacked hierarchy for activity recognition systems. Expert Syst. Appl..

[73] ud din Tahir S.B., Jalal A., Kim K. (2020). Wearable Inertial Sensors for Daily Activity Analysis Based on Adam Optimization and the Maximum Entropy Markov Model. Entropy.

[74] Ha S., Yun J.M., Choi S. Multi-modal Convolutional Neural Networks for Activity Recognition. Proceedings of the 2015 IEEE International Conference on Systems, Man, and Cybernetics.

[75] Ha S., Choi S. Convolutional neural networks for human activity recognition using multiple accelerometer and gyroscope sensors. Proceedings of the 2016 International Joint Conference on Neural Networks (IJCNN).

[76] Lyu L., He X., Law Y.W., Palaniswami M. (2017). Privacy-Preserving Collaborative Deep Learning with Application to Human Activity Recognition. Proceedings of the 2017 ACM on Conference on Information and Knowledge Management.

[77] Jalal A., Batool M., Kim K. (2020). Stochastic Recognition of Physical Activity and Healthcare Using Tri-Axial Inertial Wearable Sensors. Appl. Sci..

[78] Chowdhury A.K., Tjondronegoro D., Chandran V., Trost S.G. (2018). Physical Activity Recognition Using Posterior-Adapted Class-Based Fusion of Multiaccelerometer Data. IEEE J. Biomed. Health Inform..

